# Gendered Differences in Experiences of Bullying and Mental Health Among Transgender and Cisgender Youth

**DOI:** 10.1007/s10964-023-01786-7

**Published:** 2023-05-18

**Authors:** Laura Sares-Jäske, Mercedesz Czimbalmos, Satu Majlander, Reetta Siukola, Reija Klemetti, Pauliina Luopa, Jukka Lehtonen

**Affiliations:** 1 Finnish Institute for Health and Welfare, P.O. Box 30, FI-00271 Helsinki, Finland; 2 Åbo Akademi University, Tuomiokirkontori 3, FI-20500 Turku, Finland; 3 University of Oulu, P.O. Box 8000, FI-90014 Oulu, Finland

**Keywords:** Transgender youth, Transmasculine youth, Transfeminine youth, Bullying, Mental health, Cisgender youth

## Abstract

Even though previous studies have shown that transgender youth have poorer mental health and more experiences of being bullied than their cisgender counterparts, and that bullying associates with poorer mental health, knowledge on such associations in different gender identity groups is scarce. This study investigated how mental health problems and experiences of being bullied appear across different gender identity groups, and how bullying is associated with mental health among the groups in question. Data from the Finnish School Health Promotion 2021 study (*n* = 152,880, mean age 16.2 years (standard deviation 1.22)) was used and categorized into four gender identity groups: cisgender girls (*n* = 76,521), cisgender boys (*n* = 69,735), transfeminine youth (*n* = 1317), and transmasculine youth (*n* = 5307). Transgender youth experienced more bullying and reported poorer mental health than cisgender youth. While transfeminine youth faced the most bullying, transmasculine youth had the most mental health symptoms. In each group, bullying associated with poorer mental health. Compared to cisgender boys without bullying experiences, odds of poorer mental health were dozens-fold among transmasculine youth with weekly bullying experiences. In addition, compared to cisgender boys with bullying experiences, odds of poorer mental health were greater among all other gender identity groups with bullying experiences, and among transmasculine youth in particular (e.g., odds ratio of generalized anxiety = 8.36 (95% confidence interval, 6.59–10.6)). Bullying is associated with poorer mental health in all youth, but transgender youth, and especially transmasculine youth, may be in an even more vulnerable position for its implications. This suggests that there is a need for improving effective measures to decrease bullying in schools and to improve wellbeing of transgender youth.

## Introduction

Transgender youth are those whose gender identity does not align with the sex that they were assigned at birth, and cisgender youth are those whose gender identity does align. Previous research has shown that transgender youth are at risk of facing violence, bullying, and discrimination, often resulting from prevailing cis- and gender normative thinking, and they are more likely to report mental health challenges than their cisgender peers (Ryan & Rivers, 2003; Clark, et al., 2014; Earnshaw, et al., 2016). In addition, experiences of being bullied have been shown to associate with poorer mental health and health related quality of life (Arseneault, et al., 2010; Dubey, et al., 2022). Only a little information, however, exists on associations between bullying and mental health among groups categorized according to gender identity. The present study addressed this research gap by investigating whether the associations between experiences of being bullied and several wellbeing and mental health indicators differ between cisgender girls, cisgender boys, transfeminine youth, and transmasculine youth.

### Experiences of Being Bullied Among Transgender and Cisgender Youth

All children can be bullied, yet evidence shows that children who are perceived to be vulnerable or “different” in any way are more at risk (Cook, et al., 2010; Arseneault, 2018). Students viewed as gender non-conforming are at higher risk of bullying (Lowry, et al., 2020). School violence refers to all forms of violence that takes place in and around schools and is experienced by students and perpetrated by other students, teachers, and other school staff. Bullying is one of the most pervasive forms of school violence. It affects one’s behavioral, social, cognitive, and emotional wellbeing and is associated with absenteeism and academic failure (Menesini & Salmivalli, 2017; UNESCO, 2019). Bullying is usually defined as verbal and physical behaviors that cause another person injury or discomfort, and that are characterized by relationship-based power imbalances. Such actions are conscious, repeated, and deliberately aggressive (Mishna, 2012; Källmén & Hallgren, 2021). Bullying can take the form of physical contact, words, or more subtle actions (APA, 2022). Bullying can also be present in the form of cyberbullying, which can be defined as bullying through social media, and other information technologies (Heino, et al., 2021).

School violence that is perpetrated as a result of gender norms and stereotypes and enforced by unequal power dynamics is referred to as school-related gender-based violence. It includes in particular a specific type of gender-based violence that is linked to the actual or perceived sexual orientation and gender identity or expression of victims, including homophobic and transphobic bullying (UNESCO, 2019; Lee & Qi, 2021). Among the large body of literature focusing on sexual and gender minorities, bullying has previously been associated with stigmatization, that is, the social devaluation and discrediting of sexual and gender minorities (Hatzenbuehler & Pachankis, 2016; Abreu & Kenny, 2017; Kosciw, et al., 2020). Nevertheless, not all of the school violence the gender minority youth face is based on gender normative motives. Such experiences can also be due to other reasons, such as being “an easy target” because of, for example, loneliness, lack of social support network, or mental health problems (Lehtonen, 2021a). A large meta-analysis concluded that a bully victim is typically likely to demonstrate internalizing symptoms but also externalizing behavior, lack social skills, have negative self-related cognitions, and be noticeably rejected and isolated by peers (Cook, et al., 2010).

Generally, literature on the topic consistently shows that experiences of being bullied are more common among transgender youth than among cisgender youth (Earnshaw, et al., 2016). The definitions of “being bullied,” however, vary between studies in terms of for example the frequency of bullying, and the timeline the question inquires. Thus, the prevalence values cannot always be fully comparable. A large study comparing girls and boys with data from 40 countries found that the prevalence of being bullied at least twice during the past 2 months varied from 9 to 45% among boys, and from 5 to 36% among girls with the mean of the boys and girls being 13% (Craig, et al., 2009). Among the studies acknowledging also other gender identities than cisgender ones, an American study indicated that while 58% of cisgender boys and of cisgender girls had had any bullying experience during the past 12 months, among transgender or gender nonconforming youth the corresponding percentage value was 83% (Reisner, et al., 2015b). Another American study showed prevalence value of 15% in 2017 and in 2019 for cisgender youth and 31% in 2017 and 35% in 2019 for transgender youth of ever been bullied at school during the past 12 months (Turban, et al., 2022). In addition, results from the 2013–2015 California Student Survey indicated that 48% of the transgender participants had experienced gender-based or homophobic bullying during the past 2 months (Day, et al., 2018). Information on bullying in subgroups of gender minority youth is scarce. A large Chinese study, however, showed that 9% of cisgender boys, 5% of cisgender girls, 19% of transgender girls assigned male at birth (AMAB), 9% of transgender boys assigned female at birth (AFAB), 16% of nonbinary youth AMAB, 7% of nonbinary youth AFAB, 16% of questioning youth AMAB, and 9% of questioning youth AFAB had been bullied at school during the ongoing academic year (Wang, et al., 2020).

### Mental Health Symptoms Among Transgender and Cisgender Youth

An increasing body of research has focused on the health and wellbeing of transgender youth. Despite the inconsistence in operationalization of mental health outcomes across studies, such studies have consistently shown poorer wellbeing and more mental health problems among transgender youth than among cisgender youth (Reisner, et al., 2016). Gender minority youth are more prone to experiencing depression, anxiety, and loneliness (Verbeek, et al., 2020). According to the results of a nationally representative study from New-Zealand, the odds of significant depressive symptoms among transgender youth were almost six-fold compared to the cisgender participants (Clark, et al., 2014). Mental health symptoms at their worst, can be associated with suicidal ideation and actions. Indeed, suicidality appears to be more common among transgender youth than among cisgender youth (Grossman & D’Augelli, 2007; Clark, et al., 2014).

Recent international studies have reflected on the mental health of subgroups of transgender youth, suggesting for example that transmasculine youth have a higher prevalence of deficit and depressive disorders (Becerra-Culqui, et al., 2018), and that they appear at higher risk of suicidality compared with transfeminine, non-binary, or gender-questioning youth (Toomey, et al., 2018). In line with this, an American study of almost 81,000 participants, based on the 2016 Minnesota Student Survey, reported that mental health symptoms were more common among transmasculine youth than transfeminine youth or cisgender youth (Eisenberg, et al., 2017). Also, a more recent study, based on the 2019 Minnesota Student Survey, concluded that of the different gender identity groups, rates of depressive symptoms were the highest among nonbinary and transmasculine youth who identified their official sex as female (Gower, et al., 2022). In contrast, however, a study examining mental health differences between diverse groups of gender minority youth and cisgender sexual minority youth found that transgender women and non-binary AMAB youth reported worse mental health than transgender men and non-binary AFAB youth (Newcomb, et al., 2020). In that study though, all of the gender minority groups reported worse outcomes than cisgender sexual minority youth. Not all studies, however, have found clear differences between the groups. In a large Chinese study, no distinct conclusions could be drawn on whether transgender AFAB or AMAB youth had more suicidal behavior, but different used outcome measures showed different risks, suggesting that while transgender AFAB youth may be more subject to self-harm, transgender AMAB may be more inclined to attempt suicide (Wang, et al., 2020). In addition, an American retrospective cohort study failed to find differences between transfeminine and transmasculine groups in risks of various mental health outcomes (Reisner, et al., 2015a).

### Associations Between Bullying and Mental Health Symptoms

Bullying undermines the health of all youth and has implications for poor mental and physical health, depression, and suicidal ideation (Heikkilä, et al., 2013; Veale, et al., 2017; Abreu, et al., 2022). A large meta-analysis also found that experiences of being bullied were associated with suicidal ideation and behavior in the general youth population (Katsaras, et al., 2018). Furthermore, a previous study shows that being bullied at school and loneliness were quite clearly associated with suicidal behavior for girls, but the risk ratios were generally more than double for boys compared to girls. Boys who were bullied weekly at school had seven times the risk of a lifetime suicide attempt compared to boys who were not bullied or were bullied less often (Haravuori, et al., 2022). On the other hand, evidence has suggested that internalizing problems and depressive symptoms predicted greater later peer victimization (Kochel, et al., 2012; Vaillancourt, et al., 2013), thus potentially causing a vicious cycle between bullying and mental health problems. However, as both experiences of being bullied and mental health symptoms are common among transgender youth, it raises questions as to whether the burden of bullying is even more detrimental for them. As on average, transgender youth face more stressors in their lives than cisgender youth do -for instance gender normative thinking, body dysphoria, loneliness, and rejection by family and peers- it is likely that their resilience to cope with bullying may be lower than that of cisgender youth. A few previous studies treating gender and sexual minority youth as one group have shown cross-sectional and longitudinal associations between bullying or discrimination and greater mental distress (Birkett, et al., 2015; Earnshaw, et al., 2016). A review article of factors associated with suicidality concluded that there is a strong association between experiences of gender-based victimization or bullying and suicidality among transgender and gender-diverse youth (Bochicchio, et al., 2021). Moreover, an Australian study found that self-harm (46.2% versus 30.1%) and suicidal attempts (27.5% versus 16.1%) due to homophobic/cissexist abuse were notably more common among transgender youth than among cisgender youth with same-sex interest (Jones & Hillier, 2013). Despite the availability of such studies, those, who specifically reflect on the associations in question among subgroups of gender minority youth and compare the associations across different gender identity groups are almost nonexistent.

### Diversity of Transgender Youth in the Research

In the currently existing literature, matters concerning transgender youth have often been analyzed and discussed together with sexual minority youth, despite the differences between these two groups. It can be meaningful to compare the experiences of transgender youth to the experiences of sexual minority youth, but it might be problematic to analyze their experiences as one sexual and gender minority group. Lately, a growing part of research has focused on transgender youth specifically. Differences, however, also exist between subgroups of transgender youth, and indeed some studies have started to categorize transgender youth into various groups. In these categorizations transgender youth have often been dichotomized according to sex assigned at birth and trans/nonconforming gender identity (Eisenberg, et al., 2017), but in some studies also into more specific categorizations, such as into six categories according to sex assigned at birth and according to gender identities of transgender; nonbinary or not exclusively male or female; or questioning (Toomey, et al., 2018; Wang, et al., 2020).

### Finnish Context

In Finland, a number of studies have focused on gender and sexual minority youth. These studies suggest that in comparison to their cisgender, heterosexual counterparts, such minority youth are significantly more likely to feel stress, anxiety, and depression (Jokela, et al., 2020; Lehtonen, 2021b; Majlander, et al., 2022). In addition, previous research has shown that transgender youth face violence and the threat of it in many forms in their schools (Lehtonen, 2021a). A Finnish study utilizing data from The School Health Promotion Study wave conducted in 2017 showed that 4.3% of cisgender youth, 12.8% of those identifying as opposite sex, and 16.5% of those with non-binary gender in the comprehensive education, and 1.6% of cisgender youth, 5.8% of those identifying as opposite sex, and 8.5% of those with non-binary gender in the upper secondary education had been bullied at least once a week during the ongoing school term (Heino, et al., 2021). The results of another Finnish study showed that being subjected to bullying was associated with emotional and mental health symptoms among both heterosexual and sexual minority youth (Kurki-Kangas, et al., 2020). Another study showed that both being bullied and acting as a bully were associated with depression and suicidality (Kaltiala-Heino, et al., 1999). Little information, however, exists on the associations between experiences of being bullied and mental health in different transgender subgroups.

## Current Study

As mentioned earlier, previous research has indicated more mental health symptoms and more experiences of being bullied among transgender youth than among cisgender youth. However, information on whether the associations between bullying and poorer mental health are divergent among different gender identity groups remains scarce. Therefore, this study aimed to explore in a cross-sectional setting whether being bullied at school is associated with several poor mental health and wellbeing outcomes in different transgender (i.e., transfeminine and transmasculine) and cisgender groups (cisgirls and cisboys), and how the likelihood of poor mental health varies across groups according to bullying experiences and gender identity simultaneously. It was hypothesized that transgender youth face more bullying and have poorer mental health than cisgender youth, but also among transgender youth, bullying is even more strongly associated with mental health symptoms. Moreover, based on the previous studies, and on average different and gendered raising premises of the youth, it was expected that differences exist between transfeminine and transmasculine youth in the prevalence of the experiences of being bullied and poor mental health, as well as in the associations between bullying and mental health. It was hypothesized that transmasculine youth have more mental health problems, while transfeminine youth face more bullying.

## Methods

### Finnish School Health Promotion Study Data

This study used data from the Finnish School Health Promotion (SHP) study, conducted in March-May 2021 (Helenius, et al., 2022). The biennially implemented study monitors the wellbeing, health, and schoolwork of Finnish children and adolescents and aims to enhance the planning and evaluation of health promotion activities at school, municipal, and national levels. The data collection was conducted during a school day by an anonymous computer-administered questionnaire. All 4th, 5th, 8th and 9th graders from comprehensive school, 1st and 2nd graders from general upper secondary school, and 1st and 2nd graders from vocational upper secondary school were invited to participate. In Finland, pupils continue from the 9th grade of comprehensive school to the 1st grade in these schools, and thus the 1st grade corresponds to the 10th grade in some other school systems. In Finland, education is free, and nearly all students continue to upper secondary education from the 9-year compulsory comprehensive school. For instance, in 2021, ~93% of those graduating from comprehensive school continued to upper secondary school: 53% in general upper secondary school and 40% in vocational upper secondary school (Statistics Finland, 2022). The SHP study was approved by the ethical committee of the Finnish Institute for Health and Welfare. Parents of the students were informed of the study, and participation was voluntary.

In 2021 altogether 274,171 students participated in the study (Fig. S1). Of that sample, students answering to simplified forms (because of e.g., of a particular disability) and students at the 4th and 5th grades were excluded due to their forms not including question on gender identity. After these exclusions, a sample of 160 796 students on the 8th and 9th grades from comprehensive school and 1st and 2nd grades from general upper secondary school and from vocational upper secondary school remained encompassing 77% of the 8th and 9th graders (mean age 15.3 years), 71% of those in the 1st and 2nd grades in general upper secondary schools (mean age 17.3 years), and 34% in the corresponding grades in vocational upper secondary schools (mean age 17.5 years) in Finland. Further, the present study excluded those with missing information in key variables (gender identity; experiences of being bullied), those aged over 25 years, and those with implausible responses. The final study sample comprised 152,880 participants (95.1% of the original sample with the forms including the question on gender identity): 76,521 cisgender girls, 69,735 cisgender boys, 1317 transfeminine youth, and 5307 transmasculine youth.

Characteristics of the study sample are presented in Table 1. The mean age of the participants was 16.2 years (standard deviation 1.22). Over half of the study sample were in the 8th and 9th grades of comprehensive school, a little less than one third in general upper secondary school, and the rest in vocational upper secondary school. A slightly greater proportion of transgender youth was in comprehensive school and a slightly smaller proportion in general upper secondary school compared to cisgender youth. Low maternal education, the family’s poor socioeconomic position, and living placed outside home were more frequent among transgender youth than cisgender youth. Foreign origin was more common in transfeminine youth (11%) than in other youth (5%).

**Table 1:** Characteristics of study population (*n* = 152,880)

	*n*	All	Cisgender		Transgender		
			Cisgirls *n* = 76,521	Cisboys *n* = 69,735	Transfeminine youth *n* = 1317	Transmasculine youth *n* = 5307	*P* value^a^
*Exposure variable*							
Experiences of being bullied	152,880						
No (%)	126,069	82.5	82.0	84.4	63.3	68.4	<0.0001
Less frequently (%)	21,593	14.1	14.9	12.5	20.7	23.7	<0.0001
At least once a week (%)	5218	3.41	3.12	3.16	16.0	7.93	<0.0001
*Outcome variables*							
Average or bad self-perceived health (%)	151,732	26.0	31.9	16.3	38.4	65.0	<0.0001
Worries about own mood (%)	144,659	39.2	54.4	18.3	49.2	81.4	<0.0001
Generalized anxiety (%)	149,320	19.4	28.5	6.64	26.7	53.2	<0.0001
Depressive symptoms (%)	150,634	20.8	27.6	10.2	33.6	59.0	<0.0001
Social anxiety (%)	150,881	34.9	45.4	20.6	42.7	68.6	<0.0001
*Covariates*							
Age, years (mean (sd))	152,880	16.2 (1.22)	16.3 (1.23)	16.2 (1.19)	16.1 (1.30)	16.0 (1.27)	<0.0001
Grade	152,880						
8th and 9th of comprehensive school (%)	85,927	56.2	54.5	57.2	63.1	65.1	<0.0001
1st and 2nd of general upper secondary school (%)	46,329	30.3	34.5	26.1	23.7	25.8	<0.0001
1st and 2nd of vocational upper secondary school (%)	20,624	13.5	10.9	16.6	13.2	9.14	<0.0001
Low maternal education^b^ (%)	147,691	3.99	4.11	3.68	6.70	5.62	<0.0001
Family’s economic situation poor^c^ (%)	150,995	5.04	5.80	3.75	8.02	10.2	<0.0001
Lives placed outside home (%)	148,790	1.10	1.03	1.07	2.38	2.15	<0.0001
Foreign origin (%)	150,799	5.22	5.12	5.22	11.4	5.33	<0.0001

*n* number of observations, *sd* standard deviation

^a^*P* value for heterogeneity between groups

^b^Comprehensive school or equivalent

^c^Own conception of family’s economic situation: fairly or very poor

### Measures

#### Gender identity

Gender identity was selected to serve as a stratifying and effect-modifying variable in this study. Finnish laws identify only two genders, and thus this study refers to the legal gender that appears in the national registries by the term “official gender.” Youth aged under 18 cannot officially change their registered gender in Finland. Questions on official gender (*boy/girl*) and gender identity (*boy/girl/both/neither/it varies*) were used to form a four-class variable comprising the categories: cisgender boy (officially boy; identity boy), cisgender girl (officially girl; identity girl), transfeminine youth (officially boy; identity girl/both/neither/it varies), and transmasculine youth (officially girl; identity boy/both/neither/it varies). It is important to emphasize that both the research categories of cisgender and transgender youth include diverse respondents, and that it is not assumed that all cisgender youth in the survey would use the term cisgender to define their gender, or, that all transgender youth would use the term transgender to define their gender. The survey does not include data on what kinds of terms of self-definition the respondents used in describing their gender. In transgender groups there most likely are respondents who defined themselves as transsexual, trans, non-binary, genderless, genderqueer and so on. In the article, transfeminine and transmasculine groups are used as umbrella groups for those respondents who did not answer that their official gender would be the same as their experienced gender (gender identity), divided into two groups based on their official gender. A 10-class variable (two-category official gender * five-category gender identity) was tested and presented in Supplemental Table 1, but due to too few participants in some of the categories to enable all analyses, it was omitted from the primary analyses.

#### Experiences of being bullied

Experiences of being bullied were used as an explanatory variable. Experiences were inquired about with a question concerning bullying (“*In this questionnaire, bullying refers to the harassment of a student by another student or a group of students either verbally or physically. Teasing a student repeatedly in ways he or she does not like is also considered bullying. An argument between two roughly equal students is not considered bullying*.”) during the ongoing semester at an educational institution (*several times a week/about once a week/less frequently/not at all*). Bullying concerned all kinds of bullying and not only that due to transphobia. In Finland, the use of the term “school violence” has increased when talking about serious bullying that includes both verbal and physical bullying. In this study, however, we use the term “bullying,” (Fin. *kiusaaminen*) according to the wording in the questionnaire. For this study, a three-class variable was formed by combining two response options with the most frequent bullying (several times a week or about once a week/less frequently/not at all). In previous studies, a dichotomous variable has been used, further combining two response options with the least frequent bullying (Knaappila, et al., 2018; Heino, et al., 2021). In this study, the original four-class variable was used for sensitivity analyses.

#### Mental health and wellbeing indicators

Five outcome variables were selected to represent mental health and wellbeing: average or bad self-perceived health, worries about own mood, generalized anxiety, depressive symptoms, and social anxiety.

##### Average or bad self-perceived health

The question on self-perceived health with four response options (*very good/fairly good/average/fairly bad or very bad*) was recoded into a binary indicator variable of average or bad self-perceived health.

##### Worries about own mood

The question concerning worries about own mood during the past 12 months was used as a binary variable (*yes/no*).

##### Generalized anxiety

The anxiety indicator measuring moderate or severe generalized anxiety during the past 2 weeks was formed based on the GAD-7 (generalized anxiety disorder) measure (Spitzer, et al., 2006). The GAD-7 measure includes the following questions: “*Over the last 2 weeks, how often have you been bothered by the following problems? (1) Feeling nervous, anxious, or on edge, (2) Not being able to stop or control worrying, (3) Worrying too much about different things, (4) Having trouble relaxing, (5) Being so restless that it is hard to sit still, (6) Becoming easily annoyed or irritable, (7) Feeling afraid, as if something awful might happen*.” Response alternatives include: (1) *not at all* (0 points), (2) *several days* (1 point), (3) *more than half the days* (2 points), (4) *nearly every day* (3 points). The GAD-7 score can range from 0 to 21 as follows: 0–4: Minimal anxiety, 5–9: Mild anxiety, 10–15: Moderate anxiety, 16–21: Severe anxiety. A score of at least 10 was set as the cut-off value for a binary variable indicating moderate to severe generalized anxiety symptoms (Spitzer, et al., 2006).

##### Depressive symptoms

Depressive symptoms during the past 2 weeks were measured with the PHQ-2 screener (Patient Health Questionnaire 2) (Kroenke, et al., 2003). The PHQ-2 screener includes the following questions: “*Over the last 2 weeks, how often have you been bothered by the following problems? (1) Little interest in or little pleasure from doing various things, (2) low spirits, depression, feelings of hopelessness)*”. Response alternatives include: (1) *not at all* (0 points), (2) *several days* (1 point), (3) *more than half the days* (2 points), (4) *nearly every day* (3 points). The score can range from 0 to 6, and a score of 3–6 was defined as having depressive symptoms.

##### Social anxiety

The social anxiety indicator was based on Mini-SPIN (abbreviated version of the Social Phobia Inventory) measure (Connor, et al., 2001). In this study, the measure included three statements concerning the past week: “*Fear of embarrassment causes me to avoid doing things or speaking to people*,” “*I avoid activities in which I am the center of attention*,” and “*Being embarrassed or looking stupid are among my worst fears*.” The response options were: “*Not at all*” (0 points), “*A little bit*” (1 point), “*Somewhat*” (2 points), “*Very much*” (3 points), and “*Extremely*” (4 points). The score ranged from 0 to 12 points and a score of 6 points or more was defined as having social anxiety (Ranta, et al., 2012).

#### Sociodemographic and socioeconomic background indicators

Age, grade, low maternal education, family’s poor economic situation, living placed outside home, and foreign origin were selected to serve as background variables.

##### Age

Age was used as a continuous variable.

##### Grade

Grade was used as a three-class variable: 8th and 9th graders in comprehensive school, 1st and 2nd graders in general upper secondary school, and 1st and 2nd graders in vocational upper secondary school.

##### Low maternal education

Question on highest maternal education included the response options: *comprehensive school or equivalent; upper secondary school, high school or vocational education institution; occupational studies in addition to upper secondary school, high school or vocational education institution; and university, university of applied sciences or other higher education institution*. Comprehensive school or equivalent was chosen to represent low maternal education.

##### Family’s poor economic situation

Participants were asked to rate their family’s financial situation with the response options: *very good, fairly good, moderate, fairly poor, or very poor*. Fairly or very poor financial situation were recoded to represent the family’s poor economic situation.

##### Living placed outside home

Participants’ living situations were inquired about with a question comprising 16 residence alternatives. In this study, a binary variable was used: lives placed outside home/other.

##### Foreign origin

Questions on participants’ and their parents’ origin were used to form a binary variable with alternatives: foreign origin (both parents or the only parent with foreign origin), Finnish origin (one or both parents with Finnish origin).

### Statistical Methods

**Fig. 1:**
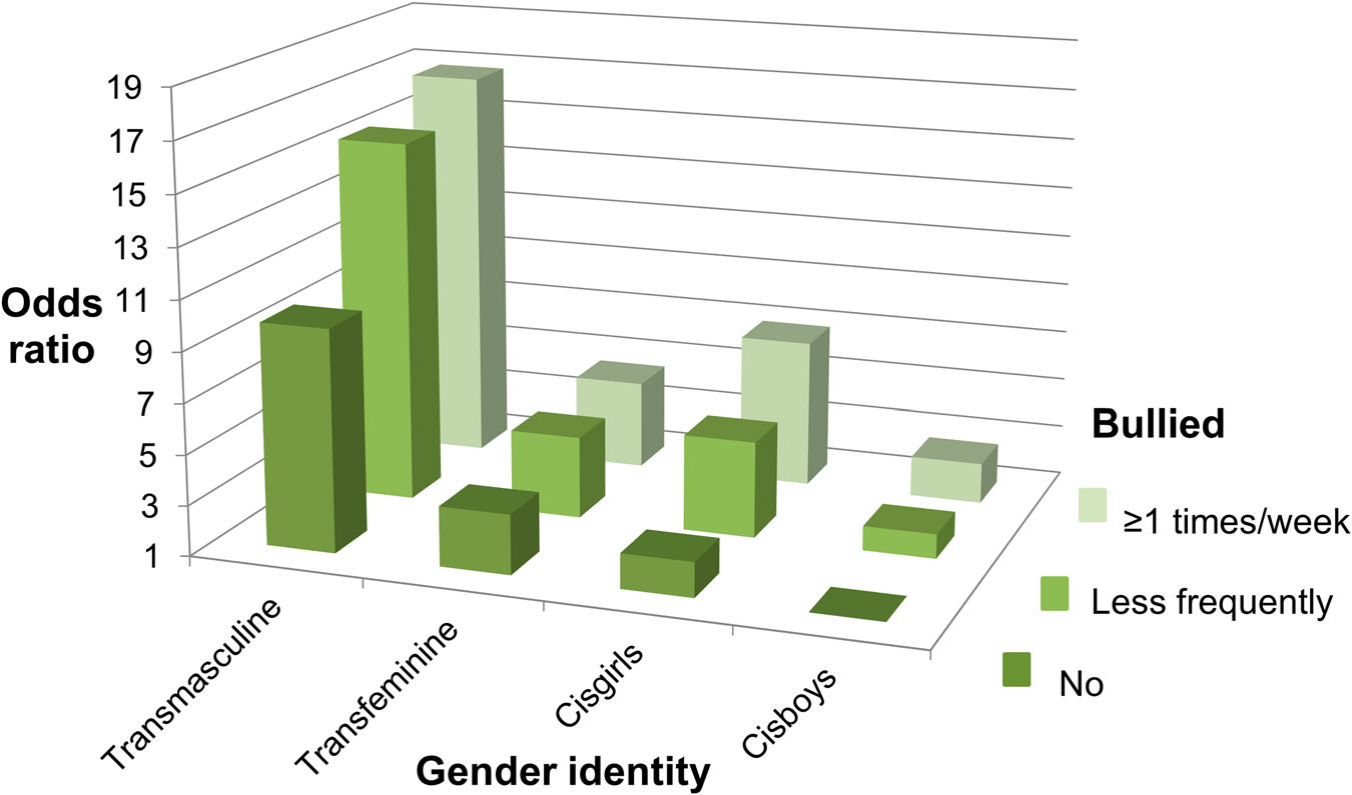
Odds of average or bad health in groups according to experiences of being bullied and gender identity (reference: not bullied, cisgender boys). All groups differed statistically significantly from the reference (*P* < 0.05). Adjusted for age and grade

**Fig. 2:**
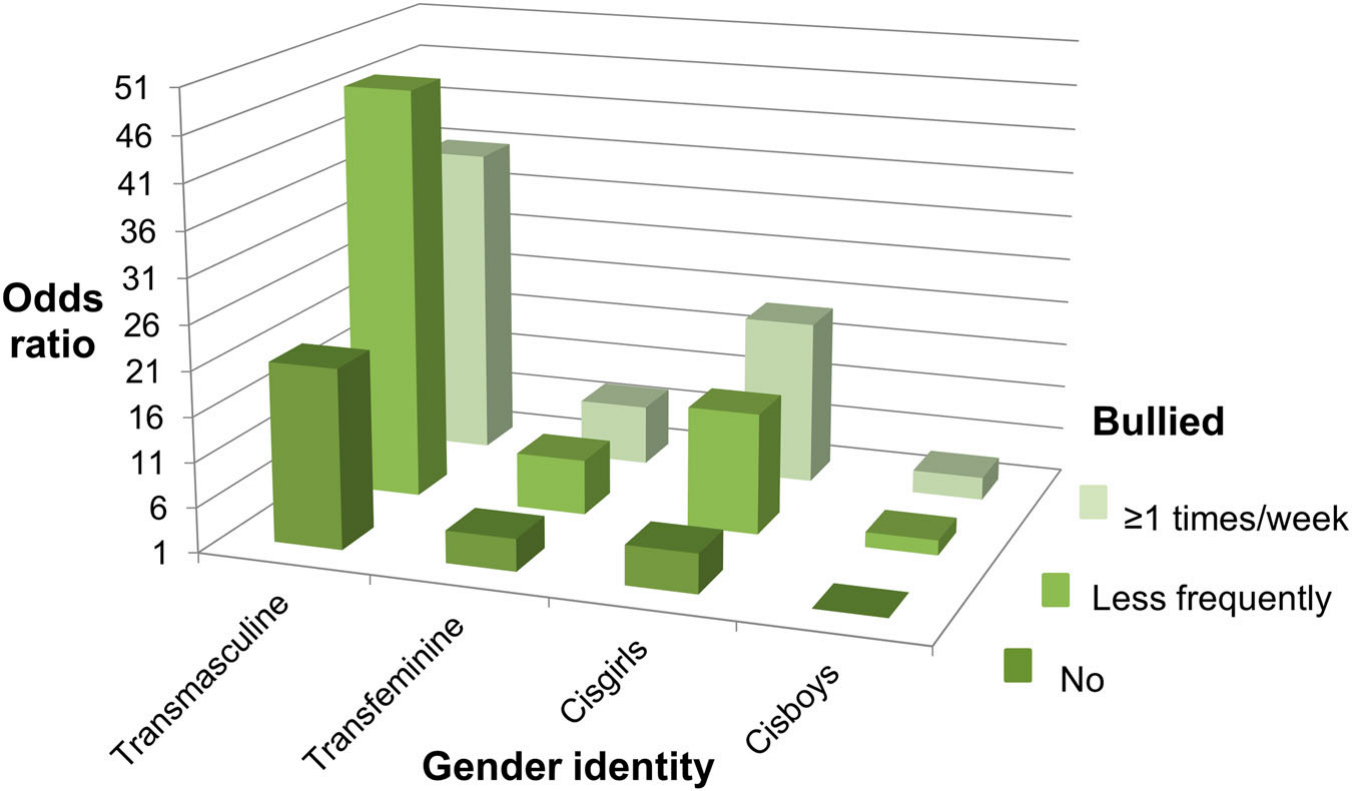
Odds of worries about mood in groups according to experiences of being bullied and gender identity (reference: not bullied, cisgender boys). All groups differed statistically significantly from the reference (*P* < 0.05). Adjusted for age and grade

**Fig. 3:**
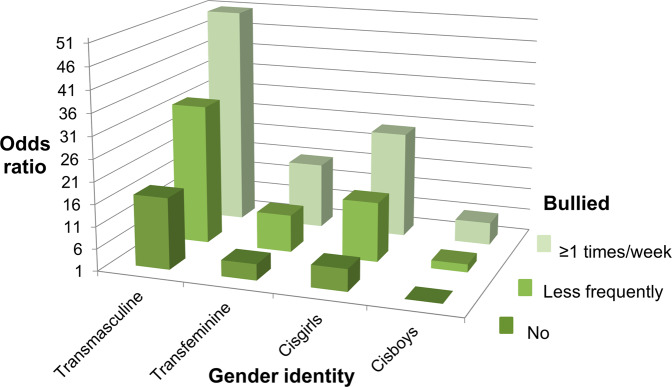
Odds of generalized anxiety in groups according to experiences of being bullied and gender identity (reference: not bullied, cisgender boys). All groups differed statistically significantly from the reference (*P* < 0.05). Adjusted for age and grade

**Fig. 4:**
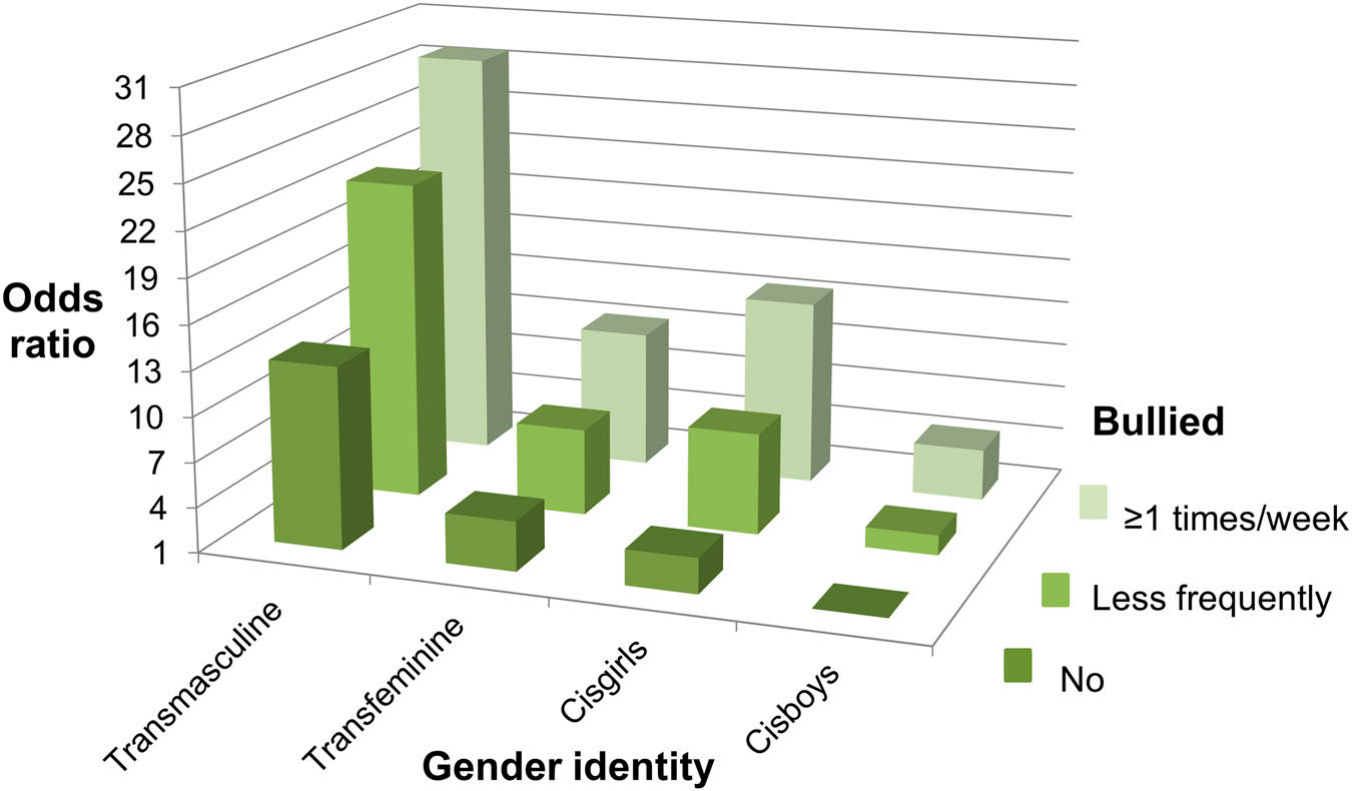
Odds of depressive symptoms in groups according to experiences of being bullied and gender identity (reference: not bullied, cisgender boys). All groups differed statistically significantly from the reference (*P* < 0.05). Adjusted for age and grade

**Fig. 5:**
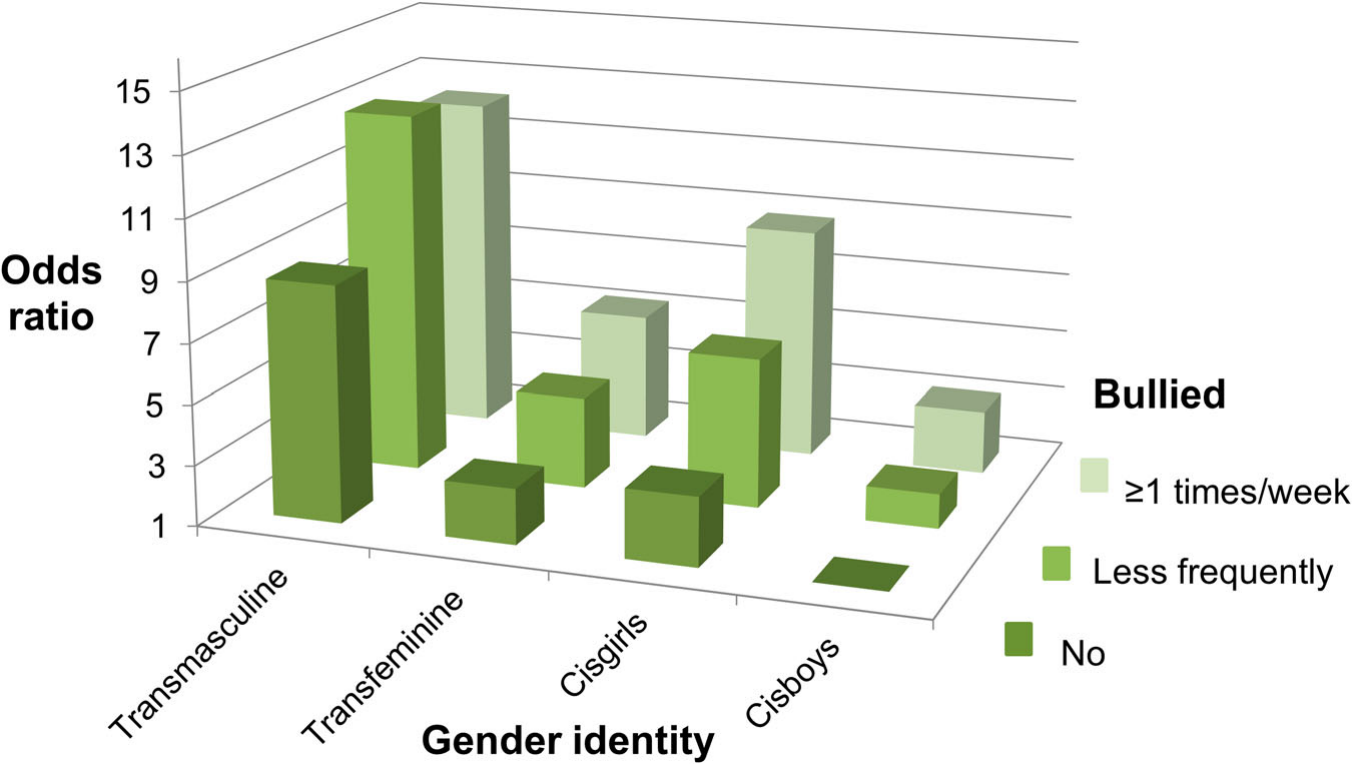
Odds of social anxiety in groups according to experiences of being bullied and gender identity (reference: not bullied, cisgender boys). All groups differed statistically significantly from the reference (*P* < 0.05). Adjusted for age and grade

Linear (Tables 1, S1, S2) and logistic (Tables 2, 3, S3, Figs. 1–5) regression models were utilized to study associations between variables of interest. The analyses were conducted for the whole study population and by using the four-class gender identity variable as a stratifying (Tables 2, S3) or an interaction (Table 3, Figs. 1–5) variable. For the interaction analyses and to study the odds of outcome indicators simultaneously between all groups, three 12-class interaction variables containing bullying and gender identity variables were formed; in each of these variables different reference categories (cisboys with 1) no, 2) less frequent, 3) at least once a week experiences of being bullied) were selected to be able to compare other gender identity groups within the bullying categories (Table 3) and across all categories (Table 3, Figs. 1–5). The strength of association was estimated with means or prevalence values and odds ratios (OR). The effect modification of gender identity in the association between experiences of being bullied and five outcome variables was studied by including in the model an interaction term between the bullying variable and the gender identity variable (P-values for gender identity interaction in Tables 2, S3).

**Table 2:** Odds^a^ of poor health according to selected indicators between categories of experiences of being bullied in gender identity groups

			Cisgender				Transgender			
Poor health indicators	All *n* = 152,880		Cisgirls *n* = 76,521		Cisboys *n* = 69,735		Transfeminine youth *n* = 1317		Transmasculine youth *n* = 5307	
	N/n	OR (95% CI)	N/n	OR (95% CI)	N/n	OR (95% CI)	N/n	OR (95% CI)	N/n	OR (95% CI)
*Average or bad self-perceived health*										
Experiences of being bullied	39475/151732		24255/76068		11293/69094		499/1299		3428/5271	
No (reference)	29133/125162	1	18031/62398	1	8561/58332	1	301/824	1	2240/3608	1
Less frequently	8070/21426	2.10 (2.04–2.17)	4986/11313	2.00 (1.92–2.09)	2088/8598	2.03 (1.92–2.14)	111/270	1.28 (0.96–1.70)	885/1245	1.56 (1.35–1.80)
At least once a week	2272/5144	2.79 (2.63–2.95)	1238/2357	2.83 (2.61–3.08)	644**/**2164	2.70 (2.45–2.97)	87/205	1.36 (0.99–1.87)	303**/**418	1.69 (1.35–2.13)
P for heterogeneity		<0.0001		<0.0001		<0.0001		0.08		<0.0001
P for gender identity interaction										<0.0001
*Worries about own mood*										
Experiences of being bullied	56739/144659		40042/73559		11847/64655		609/1237		4241/5208	
No (reference)	42276/118904	1	30490/60152	1	8625/54417	1	361/783	1	2800/3552	1
Less frequently	11502/20734	2.61 (2.53–2.69)	7797/11072	2.64 (2.52–2.76)	2474/8162	2.78 (2.64–2.94)	138/257	1.56 (1.16–2.09)	1093/1243	2.23 (1.84–2.70)
At least once a week	2961/5021	3.14 (2.96–3.33)	1755/2335	3.51 (3.19–3.87)	748/2076	3.72 (3.38–4.10)	110/197	1.68 (1.21–2.33)	348/413	1.69 (1.27–2.24)
P for heterogeneity		<0.0001		<0.0001		<0.0001		0.0007		<0.0001
P for gender identity interaction										<0.0001
*Generalized anxiety*										
Experiences of being bullied	28967/149320		21358/74861		4515/68008		338/1267		2756/5184	
No (reference)	20153/123285	1	15320/61442	1	2964/57480	1	164/811	1	1705/3552	1
Less frequently	6652/21004	2.57 (2.48–2.66)	4751/11106	2.39 (2.29–2.49)	1055/8414	2.97 (2.75–3.20)	84/257	2.16 (1.57–2.98)	762/1227	1.85 (1.61–2.12)
At least once a week	2162/5031	4.29 (4.04–4.55)	1287/2313	4.10 (3.77–4.46)	496/2114	6.47 (5.80–7.22)	90/199	3.60 (2.54–5.11)	289/405	2.84 (2.26–3.57)
P for heterogeneity		<0.0001		<0.0001		<0.0001		<0.0001		<0.0001
P for gender identity interaction										<0.0001
*Depressive symptoms*										
Experiences of being bullied	31378/150634		20822/75526		7024/68566		435/1293		3097/5249	
No (reference)	22073/124257	1	14927/61979	1	4939/57871	1	236/822	1	1971/3585	1
Less frequently	7027/21264	2.41 (2.33–2.49)	4622/11207	2.27 (2.17–2.37)	1476/8543	2.48 (2.33–2.65)	101/267	1.67 (1.24–2.24)	828/1247	1.66 (1.44–1.91)
At least once a week	2278/5113	3.99 (3.77–4.23)	1273/2340	3.90 (3.58–4.24)	609/2152	4.78 (4.33–5.29)	98/204	2.53 (1.82–3.51)	298/417	2.09 (1.67–2.63)
P for heterogeneity		<0.0001		<0.0001		<0.0001		<0.0001		<0.0001
P for gender identity interaction										<0.0001
*Social anxiety*										
Experiences of being bullied	52674/150881		34393/75755		14113/68564		554/1297		3614/5265	
No (reference)	39747/124513	1	26491/62175	1	10544/57909	1	320/825	1	2392/3604	1
Less frequently	10125/21264	2.01 (1.95–2.07)	6370/11239	1.78 (1.71–1.85)	2709/8512	2.24 (2.13–2.36)	125/267	1.55 (1.16–2.06)	921/1246	1.41 (1.22–1.64)
At least once a week	2802/5104	2.73 (2.58–2.89)	1532/2341	2.59 (2.37–2.82)	860/2143	3.26 (2.98–3.57)	109/205	2.04 (1.48–2.80)	301/415	1.31 (1.04–1.65)
P for heterogeneity		<0.0001		<0.0001		<0.0001		<0.0001		<0.0001
P for gender identity interaction										<0.0001

*CI* confidence interval, *n* individuals in the category, *N* cases in the category, *OR* odds ratio

^a^Adjusted for age (continuous) and grade

**Table 3:** Odds of poor health according to selected indicators between interaction categories of experiences of being bullied and gender identity^a^

	Cisgender		Transgender	
Poor health indicators^b^	Cisgirls OR (95% CI)	Cisboys OR (95% CI)	Transfeminine youth OR (95% CI)	Transmasculine youth OR (95% CI)
***Average or bad self-perceived health***				
*Reference group: cisboys with no bullying experiences*				
Experiences of being bullied				
No (reference)	2.39 (2.32–2.46)	1	3.37 (2.92–3.89)	9.81 (9.13–10.5)
Less frequently	4.83 (4.63–5.05)	1.97 (1.87–2.08)	4.27 (3.34–5.45)	15.5 (13.6–17.5)
At least once a week	6.90 (6.34–7.50)	2.61 (2.38–2.88)	4.51 (3.41–5.96)	16.8 (13.5–20.9)
*Reference group: cisboys with less frequent bullying experiences*				
Experiences of being bullied				
Less frequently (reference)	2.45 (2.30–2.61)	1	2.16 (1.69–2.78)	7.84 (6.86–8.95)
*Reference group: cisboys with bullying experiences at least once a week*				
Experiences of being bullied				
At least once a week (reference)	2.64 (2.33–2.98)	1	1.72 (1.29–2.31)	6.43 (5.09–8.13)
***Worries about own mood***				
*Reference group: cisboys with no bullying experiences*				
Experiences of being bullied				
No (reference)	5.40 (5.25–5.55)	1	4.60 (3.98–5.31)	21.0 (19.3–22.9)
Less frequently	14.5 (13.8–15.2)	2.67 (2.54–2.82)	7.15 (5.57–9.19)	47.1 (39.6–56.0)
At least once a week	19.4 (17.6–21.4)	3.55 (3.23–3.90)	7.69 (5.76–10.3)	35.9 (27.5–47.0)
*Reference group: cisboys with less frequent bullying experiences*				
Experiences of being bullied				
Less frequently (reference)	5.42 (5.09–5.77)	1	2.68 (2.08–3.45)	17.6 (14.7–21.0)
*Reference group: cisboys with bullying experiences at least once a week*				
Experiences of being bullied				
At least once a week (reference)	5.47 (4.80–6.24)	1	2.17 (1.60–2.93)	10.1 (7.63–13.4)
***Generalized anxiety***				
*Reference group: cisboys with no bullying experiences*				
Experiences of being bullied				
No (reference)	6.01 (5.76–6.26)	1	4.65 (3.90–5.54)	17.2 (15.9–18.5)
Less frequently	14.5 (13.7–15.3)	2.82 (2.61–3.04)	9.49 (7.31–12.3)	32.7 (29.0–37.0)
At least once a week	25.0 (22.9–27.4)	6.08 (5.46–6.77)	15.9 (12.0–21.1)	50.8 (40.8–63.3)
*Reference group: cisboys with less frequent bullying experiences*				
Experiences of being bullied				
Less frequently (reference)	5.15 (4.78–5.54)	1	3.37 (2.58–4.40)	11.6 (10.2–13.3)
*Reference group: cisboys with bullying experiences at least once a week*				
Experiences of being bullied				
At least once a week (reference)	4.12 (3.61–4.69)	1	2.61 (1.94–3.52)	8.36 (6.59–10.6)
***Depressive symptoms***				
*Reference group: cisboys with no bullying experiences*				
Experiences of being bullied				
No (reference)	3.35 (3.24–3.47)	1	4.30 (3.69–5.02)	13.1 (12.2–14.1)
Less frequently	7.76 (7.40–8.14)	2.34 (2.19–2.49)	6.78 (5.29–8.69)	22.3 (19.8–25.2)
At least once a week	13.4 (12.3–14.7)	4.44 (4.03–4.90)	10.2 (7.74–13.5)	28.7 (23.1–35.5)
*Reference group: cisboys with less frequent bullying experiences*				
Experiences of being bullied				
Less frequently (reference)	3.32 (3.10–3.55)	1	2.90 (2.25–3.73)	9.55 (8.38–10.9)
*Reference group: cisboys with bullying experiences at least once a week*				
Experiences of being bullied				
At least once a week (reference)	3.03 (2.67–3.43)	1	2.30 (1.72–3.08)	6.45 (5.11–8.13)
***Social anxiety***				
*Reference group: cisboys with no bullying experiences*				
Experiences of being bullied				
No (reference)	3.28 (3.20–3.37)	1	2.84 (2.47–3.27)	8.81 (8.19–9.47)
Less frequently	5.96 (5.71–6.22)	2.15 (2.04–2.26)	4.05 (3.18–5.15)	13.0 (11.5–14.8)
At least once a week	8.74 (8.00–9.54)	3.11 (2.84–3.40)	5.25 (3.98–6.91)	12.2 (9.86–15.2)
*Reference group: cisboys with less frequent bullying experiences*				
Experiences of being bullied				
Less frequently (reference)	2.78 (2.62–2.95)	1	1.88 (1.48–2.41)	6.07 (5.30–6.94)
*Reference group: cisboys with bullying experiences at least once a week*				
Experiences of being bullied				
At least once a week (reference)	2.81 (2.49–3.18)	1	1.69 (1.27–2.25)	3.94 (3.13–4.98)

*CI* confidence interval, *OR* odds ratio

^a^Adjusted for age (continuous) and grade

^b^Results of the whole model have been presented for the first comparison where cisboys with no bullying experiences have been used as a reference group. For the other comparisons, only the results of the four gender identity groups in the same bullying category as the reference group have been presented

In the logistic models, two adjustment models were used. In Model 1, age as a continuous variable and grade were adjusted for (Tables 2, 3, Figs. 1–5). In Model 2, maternal education (low/other), family’s economic situation (poor/other), living situation (placed outside home/other), and origin (foreign/other) were additionally included in the model (Table S3). All analyses were conducted using SAS Enterprise Guide, version 7.15 HF7 (SAS Institute Inc., Cary, NC, USA).

## Results

### Experiences of Being Bullied Among Transgender and Cisgender Youth

Of the whole study population, 3.4% had experienced weekly or more frequent bullying, and additionally 14.1% had experienced less frequent bullying during the ongoing semester (Table 1). Bullying experiences were most common in transfeminine youth, but transmasculine participants also reported more common bullying experiences than cisboys or cisgirls; approximately one-third of transgender youth had been bullied weekly or less frequently during the ongoing semester. A further scrutiny with a 10-class gender identity variable showed that experiences of being bullied appeared more common in each transfeminine subgroup compared to cisgender youth or transmasculine subgroups (Table S1). However, small variation also existed between transfeminine groups, and the group of transfeminine youth with the identity of both a boy and a girl reported most frequently weekly bullying. Regarding the school levels, weekly bullying was most common in comprehensive school (5.0%) followed by vocational upper secondary school (2.4%), and least common in general upper secondary school (1.0%) (Table S2). In each school level, transfeminine respondents experienced weekly bullying most often.

### Mental Health Symptoms Among Transgender and Cisgender Youth

All five outcome indicators of poorer mental health and wellbeing showed a steep gradient across gender identity groups; transmasculine students had the worst mental health, followed by transfeminine youth and cisgirls, and lastly cisboys had the best self-reported mental health according to each indicator (Table 1). For instance, while 18% of cisboys had had worries about their own mood during the past 12 months, the corresponding percentage value for the transmasculine participants was 81%. In a more detailed analysis of 10 gender identity categories, all poor mental health indicators showed higher percentage values in all transmasculine subgroups—with a tendency toward the highest values in the nonbinary group—compared to the other subgroups (Table S1). When looking at the whole study population, respondents in general upper secondary school tended to have more poor mental health symptoms than those in the other school levels (Table S2). Across all school levels, however, transmasculine youth had the most frequent and cisboys the least frequent symptoms of poor mental health, according to each indicator. However, within gender identity groups, variation existed between indicators in terms of which school level the symptoms were most common. For instance, in transmasculine youth, while worries about own mood were most common in general upper secondary school (86%) and the least common in vocational upper secondary school (79%) (*P* for difference = 0.01), no statistically significant differences existed in generalized anxiety between three school levels.

### Gender Identity-Stratified Associations Between Bullying and Mental Health Symptoms

In the whole study population and within each gender identity group, the odds of poorer mental health according to each indicator (average or bad self-perceived health, worries about own mood, generalized anxiety, depressive symptoms, and social anxiety) increased in parallel with bullying categories after adjustment for age and grade (Table 2). For instance, in the whole study population, those who had experienced weekly bullying had over fourfold odds of generalized anxiety compared to those who had not been bullied. Accordingly, the odds of generalized anxiety were multiple in cisgirls (odds ratio (OR), 4.10; 95% confidence interval (CI), 3.77–4.46), cisboys (OR, 6.47; 95% CI, 5.80–7.22), transfeminine (OR, 3.60; 95% CI, 2.54–5.11), and transmasculine (OR, 2.84; 95% CI, 2.26–3.57) respondents with weekly bullying experiences compared to corresponding respondents who had not been bullied. In relation to all mental health indicators, the odds tended to increase more steeply across bullying categories in cisboys and cisgirls than in transfeminine and transmasculine participants (*P* value for interaction in all comparisons <0.0001) (Table 2). After additional adjustment for maternal education, family’s economic situation, living situation, and origin, the results were partly slightly attenuated but remained at a similar level (Table S3).

### Interactions Between Gender Identity, Bullying and Mental Health Symptoms

Interaction analyses comparing the odds of poorer mental health between interaction categories of experiences of being bullied and gender identity revealed that, compared to the reference category of cisboys without bullying experiences, the odds of each poor mental health indicator increased to multiple in other interaction groups and to dozens-fold in the group of transmasculine youth with weekly bullying experiences (Table 3, Figs. 1–5). For instance, transmasculine youth who had been bullied weekly had odds of 50.8 (95% CI, 40.8–63.3) of generalized anxiety compared to the reference group (Fig. 3). When having worries about their own mood or social anxiety as an outcome, transmasculine youth with less frequent experiences of being bullied also reached a similar level of odds as transmasculine youth with weekly bullying experiences. However, only 0.28% (*n* = 421) of participants belonged to the group of transmasculine youth with weekly experiences of being bullied and 0.14% (*n* = 211) to the group of transfeminine youth with weekly experiences of being bullied.

Odds of poorer mental health between interaction categories of experiences of being bullied and gender identity were also studied using reference categories of cisboys with less frequent bullying experiences and cisboys with bullying experiences at least once a week (Table 3). The odds of poorer mental health according to each indicator were greater in other gender identity groups than in cisboys in the same category of bullying experiences, being the highest in transmasculine youth. For instance, transmasculine youth with less frequent bullying experiences had odds of 11.6 (95% CI, 10.2–13.3) of generalized anxiety compared to cisboys with the same level of bullying experiences, and transmasculine youth with bullying experiences at least once a week had odds of 8.36 (95% CI, 6.59–10.6) of generalized anxiety compared to cisboys with the same level of bullying experiences.

### Sensitivity Analyses

In order to study whether the four-class gender identity variable used in the primary analyses represented more diverse gender identity subgroups, analyses with a 10-class gender identity variable were conducted (Table S1). Results of these analyses showed in accordance with the four-class variable that experiences of being bullied appeared more common in each transfeminine subgroup compared to other gender identity subgroups, and all poor mental health indicators showed higher percentage values in all transmasculine subgroups compared to the other subgroups. Small sample size in part of the categories, however, prevented the use of the 10-category variable in all of the analyses. In addition, to study the robustness of the explanatory bullying variable, sensitivity analyses as replication of the analyses of Table 2, but using the original four-class bullying variable (Bullied: several times a week/about once a week/less frequently/not at all) were conducted. According to these results, the odds in the categories of “once a week” and “several times a week” were aligned and did not statistically significantly differ from each other (data not shown). In part of these analyses, groups of transfeminine or transmasculine youth with experiences of being bullied several times a week, did not differ statistically significantly from the reference groups due to too few participants in the categories in question.

## Discussion

As numerous studies have shown, transgender youth face more bullying and have greater likelihood of mental health problems than their cisgender peers (Ryan & Rivers, 2003; Clark, et al., 2014; Earnshaw, et al., 2016). Moreover, evidence indicates an association between experiences of being bullied and poorer mental health (Arseneault, et al., 2010; Dubey, et al., 2022). However, information on associations between bullying and mental health among different gender identity groups has been lacking. Accordingly, the aims of this study were to examine whether the associations between experiences of being bullied and several wellbeing and mental health indicators are different in cisgender girls, cisgender boys, transfeminine youth, and transmasculine youth. Thus, ultimately this study strived to increase evidence of, whether bullying may be even more detrimental to transgender youth -who often face also other stressors in their lives- than to cisgender youth. The findings of this study confirmed the hypotheses of transgender youth having more experiences of being bullied and higher likelihood of poorer mental health. Even though bullying appeared to associate with poorer mental health among each gender identity group, the findings indicated that among youth who had been bullied, especially transmasculine youth had high likelihood of poorer mental health.

### Experiences of Being Bullied and Mental Health Symptoms Among Transgender and Cisgender Youth

In line with the literature, this study found that transgender youth had more experiences of being bullied than cisgender youth (Wang, et al., 2020; Lehtonen, 2021a; Turban, et al., 2022). In this study, especially transfeminine youth experienced frequent bullying. Consistent with these findings, a Chinese study of 12 108 participants with a mean age of 15.8 years showed that trans, nonbinary, and questioning youth AMAB experienced more bullying than trans, nonbinary, and questioning youth AFAB or cisgender youth (Wang, et al., 2020).

In accordance with the previous findings, transgender youth had more frequent symptoms of poor mental health than cisgender youth (Reisner, et al., 2015a; Wang, et al., 2020; Bochicchio, et al., 2021). Of this diverse group, transmasculine youth appeared to have the most symptoms. Some literature exists on subgroups of transgender youth, most of them showing in accordance with the present findings a higher prevalence of different mental health symptoms among transgender youth AFAB than among cisgender youth or transgender youth AMAB (Eisenberg, et al., 2017; Becerra-Culqui, et al., 2018; Toomey, et al., 2018). In contrast, however, some of the studies have indicated more mental health symptoms among transgender youth AMAB than among transgender youth AFAB (Newcomb, et al., 2020), or have failed to find clear differences between the groups (Reisner, et al., 2015a; Wang, et al., 2020).

### Gender Identity-Stratified Associations Between Bullying and Mental Health Symptoms

The results of this study indicated that all youth who had experienced bullying had poorer wellbeing and mental health than youth who had not experienced bullying. Several previous studies support these findings. For example, conclusions of a review article investigating bullying victimization and mental health indicated that bullying is independently associated with severe mental health symptoms in childhood and adolescence (Arseneault, et al., 2010). In line with the findings of this study, few previous studies have also found an explicit association between experiences of being bullied and poorer mental health among transgender and gender-diverse populations. A recent systematic review concluded that suicidal behavior among transgender and gender-diverse youth is associated with bullying (Bochicchio, et al., 2021). Contrary to our initial hypotheses, however, the present findings, that treated gender minority groups separately, showed a stronger direct gradient of poorer mental health across categories of being bullied in cisgender youth than in transgender youth. This may be due to the fact that mental health problems are also more frequent in the reference groups (those not being bullied) among transgender youth than among cisgender youth, which leads to smaller mental health differences between the transgender groups. Conversely among cisgender youth with no experiences of being bullied, mental health problems are less common, and compared to them, cisgender youth who have been bullied comprise a more distinct group in terms of mental health.

### Interactions Between Gender Identity, Bullying and Mental Health Symptoms

Compared to cisboys who had not experienced bullying, transmasculine youth with weekly experiences of bullying had strikingly multiple likelihood of poor wellbeing and mental health symptoms. In addition, compared to cisboys who had been bullied, all other gender identity groups with bullying experiences, and transmasculine youth in particular, had greater odds of poorer mental health. No previous studies have compared the odds of poor mental health between interaction categories of gender identity and experiences of being bullied. Thus, results of this study provide new information on the issue and highlight the significance of notable differences between the studied groups, emphasizing especially differences between transfeminine and transmasculine youth.

The present findings suggest that even though bullying is associated with poorer wellbeing and mental health in all youth, for transgender, and for transmasculine youth, in particular, bullying may be even more detrimental. When comparing cisgender boys and transmasculine youth with less frequent (than at least once a week) bullying experiences, the odds of poorer mental health in transmasculine youth were higher than what could be seen in the respective comparison of cisgender boys and transmasculine youth who experienced bullying at least once a week. This could be due to the possibility that cisgender boys who, on average, have less stressors in their life and less mental health problems, have higher resilience to occasional bullying, while for transmasculine youth, already occasional bullying can be overloading leading to mental health symptoms. This is in line with the present findings according to which more frequent weekly bullying did not increase the odds of worries about own mood or social anxiety among transmasculine youth. Instead, among transmasculine youth, less frequent bullying showed higher or rather similar odds of poorer mental health than weekly bullying. On the other hand, in separate analyses of the cisboys and cisgirls, more frequent bullying increased the odds of poorer mental health according to each indicator, suggesting a more linear gradient between bullying and poorer mental health.

### Potential Explanations for the Differences in Experiences of Being Bullied and Mental Health Symptoms Between Gender Identity Groups

There clearly are differences between transmasculine and transfeminine respondents and between cisgender groups, as well as between transgender and cisgender respondents. Transgender respondents experience bullying and mental health problems more than cisgender youth. This is in line with earlier research, demonstrating that heteronormative practices within education and minority stress attached to them are causing lower mental health. It also partially explains the possibility and interest in bullying based on non-normative gender identity or expression. A review article concluded that lower mental health among transgender youth is largely explained by stigma, lack of gender identity affirmation, and a generally non-accepting environment (Turban & Ehrensaft, 2018). It is possible that in addition to an external non-supporting environment, increased mental health symptoms are also driven by internal gender dysphoria per se. Transgender youth may have also been forced to reflect on their identity and wellbeing more than cisgender youth do, which may also lead to recognizing and reporting problems more easily in an anonymous survey such as this, where there is no fear of unsupportive confrontation.

In this study, transgender youth reported a higher prevalence of low maternal education, family’s poor socioeconomic position, and living placed outside home. In addition, transfeminine youth more frequently reported foreign origin. These factors can also play a role in the wellbeing of youth. On the other hand, it is possible that difficulties in life have impacted on reflecting on one’s gender identity. In addition to the effect of socioeconomic premises, transgender identity may hinder seeking aid and services if an adolescent does not feel that they will benefit from it, or fears not being confronted the right way.

It seems that in some ways responses of transmasculine youth, concerning mental health, resemble those of cisgirls, and vice versa, the responses of transfeminine youth resemble those of cisboys. Transmasculine youth had higher prevalence of poorer mental health according to different indicators than transfeminine youth, while similarly cisgirls had higher prevalence than cisboys. One reason for this could be that cisboys and transfeminine youth have usually been brought up as and were expected to be boys, and to show traits and characteristics that are traditionally regarded as “masculine”, and cisgirls and transmasculine youth have been brought up and were expected to be what is traditionally viewed as “feminine”. The surrounding culture has given them different opportunities to express their feelings based on their expected gender, or the official sex assigned to them at birth; boys are generally expected to experience more but suffer less from violence than girls, and girls can express their sadness and joy more than boys. Boys are more harshly judged than girls when breaking traditional gender rules (Meyer, 2009). Consequentially, those who are perceived as boys are likely to experience the same. Therefore, cisboys and transfeminine youth may find it more difficult to seek help for their problems but also to report problems in a survey such as this. In society and in many school settings, masculine features in a presumed girl are generally more acceptable than feminine features in a presumed boy (Meyer, 2009; Tähkä, 2022), which probably explains part of the higher frequency of bullying among transfeminine youth (Ristori & Steensma, 2016).

The underlying cause of mental health symptoms of transgender youth may also be anxiety due to body dysphoria. In the cases of transmasculine and nonbinary adolescents, chest dysphoria is associated with higher anxiety and depression—the association, however, is independent of the level of gender dysphoria, the degree of appearance congruence, and social transition status (Sood, et al., 2021). Cisgirls and transmasculine youth may reach puberty on average earlier than cisboys and transfeminine youth, which may explain the differences partly because of earlier psychological maturation and partly because of earlier gender dysphoria due to changes in the body.

### Strengths and Weaknesses

The strengths of this study include the large and unique youth population sample that enabled the study of transgender youth and, further, the study of more diverse gender identity groups. Most of the previous studies have examined these associations using one or two transgender youth groups, not having been able to compare them with cisgender youth or having had notably smaller data sets.

Some weaknesses also exist. First, in self-administrated youth data, there tends to be a certain proportion of mischievous responding (Robinson-Cimpian, 2014). However, an attempt was made to exclude such respondents based on their unplausible answers to questions concerning, e.g., functional capacity (Helenius, et al., 2022). A limitation with the data and analysis has to do with the fact that in the survey there are no questions on how transgender youth expressed their non-normative gender or gender identity in their schools. This is relevant when thinking about the experiences of bullying. It is easier to bully someone based on their non-normative gender expression or identity if there is an awareness of this. In addition, the survey did not specify whether the bullying was related to the person being transgender or gender nonconforming. This way it is not possible to know if the violence was transphobic violence or related to other issues or grounds. It is likely that those youth who transgress the normative gender expectations are more likely to be victims of violence and then also more likely to be victims of transphobic violence. However, some transgender youth who question gender norms might also be assumed to be non-heterosexual, resulting in them experiencing homophobic bullying.

Transgender youth face, on average, more stressors in their lives than cisgender youth, and thus, the differences in associations found in this study may also be due to other factors besides bullying, that could not be adjusted for in this study. Even though adjustment of sociodemographic and socioeconomic factors in this study did not attenuate the results notably, the possibility of residual confounding cannot be fully excluded. Moreover, no causal relationships can be derived from observational cross-sectional data, and the directions of the associations found remain unresolved. Previous studies, however, have indicated that mental health symptoms can both precede and follow bullying (Le, et al., 2019). The data and analysis in this study do not give us the chance to know whether the bullying caused mental health problems, whether mental health problems were causing bullying, or were used to justify violence. It is likely that all can be relevant when thinking of the relationships between bullying and mental health issues. Also, loneliness, which is clearly more likely for transgender youth compared to cisgender youth, can be a relevant factor that is related to both experiences of bullying and mental health issues. It is easier to choose a lonely person without support networks and friends as a victim of violence compared to a person who has friends to support them. This support and friends can also be a vital aspect in better mental health and vice versa. These three aspects are related and can create a vicious circle in which more experiences of violence, more loneliness, and more mental health problems are linked and enhance each other. Prevention of violence, friends, and a better support system for overcoming mental health problems could create a circle in which they support each other. In addition, enhancing knowledge among personnel at schools and among parents on gender diversity and on the prevention of bullying and mental health problems could help to turn the direction of the cycle from vicious to positive.

## Conclusion

Although previous research has shown higher prevalence of experiences of being bullied and mental health symptoms among transgender youth than among cisgender youth, and that in general youth populations bullying is associated with poorer wellbeing and mental health, evidence on associations between bullying and mental health in different gender identity groups has been scarce. This study aimed to examine how mental health problems and experiences of being bullied distribute across gender identity groups, and how bullying is associated with mental health among the groups in question. In accordance with the literature, the present findings highlight significant differences in experiences of being bullied and in mental health symptoms between cisgirls, cisboys, transfeminine youth, and transmasculine youth. While transfeminine youth experienced the most bullying and transmasculine youth had the most mental health symptoms, cisboys reported the least of both. Even though bullying was associated with poorer mental health in each gender identity group, compared to cisgender boys with or without bullying experiences, other gender identity groups with bullying experiences, and especially transmasculine youth, had greater odds of poorer mental health. Transgender youth should not be considered as a uniform population group, but heterogeneity exists among the group as well. This should be acknowledged when studying transgender youth and targeting policies to improve their wellbeing. Further intersectional scrutiny, including other characteristics besides gender identity (e.g., sexual orientation, origin, family socioeconomic position, and form of residence), is also needed. Bullying is associated with poorer mental health in all youth, but transgender youth may be in especially vulnerable position for its implications. The findings of this study emphasize the importance of investing in the promotion of wellbeing among transgender youth and improving inclusive bullying prevention programs.

## Supplementary Information

Supplementary material
