# Supplementary material for: Gendered Differences in Experiences of Bullying and Mental Health Among Transgender and Cisgender Youth
Source: J Youth Adolesc. 2023 May 18;52(8):1531–48. doi: 10.1007/s10964-023-01786-7 (PMC10276116; doi:10.1007/s10964-023-01786-7)
Supplement: Supplementary file 1 — Supplementary material [file 10964_2023_1786_MOESM1_ESM.docx]

Supplementary material

**Gendered Differences in Experiences of Bullying and Mental Health Among Transgender and Cisgender Youth**

Journal of Youth and Adolescence

**Fig. S1** Flow chart of the study population


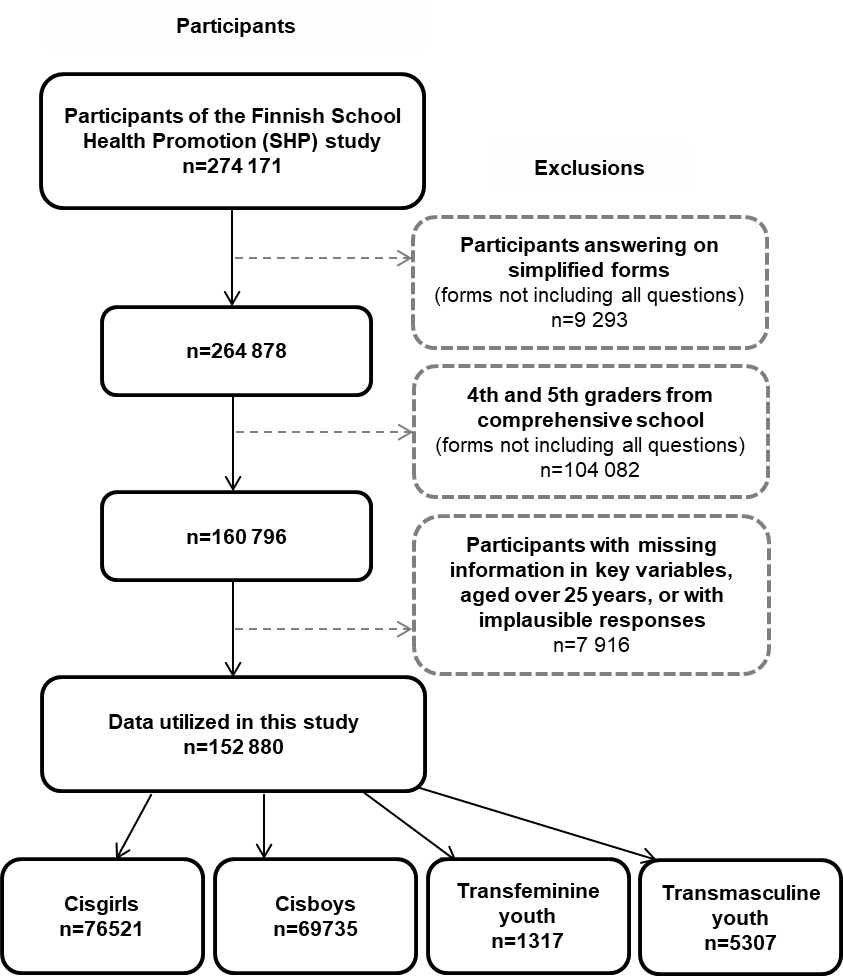


| **Table S1**. Proportions (%) of those being bullied weekly or having poor mental health according to six selected indicators in categories according to gender identity (n= 152 880). | | | | | | | |
| --- | --- | --- | --- | --- | --- | --- | --- |
| **Gender identity** | **n** | **Bullied weekly (%)** | **Average or bad self-perceived health (%)** | **Worries about own mood (%)** | **Generalized anxiety**  **(%)** | **Depressive symptoms (%)** | **Social anxiety (%)** |
| Cisgirls | 76521 | 3.12 | 31.9 | 54.4 | 28.5 | 27.6 | 45.4 |
| Cisboys | 69735 | 3.16 | 16.3 | 18.3 | 6.64 | 10.2 | 20.6 |
| Transgirls: identity girl | 256 | 11.7 | 41.7 | 58.8 | 28.9 | 36.1 | 49.4 |
| Transgirls: identity both | 185 | 20.0 | 36.4 | 50.0 | 29.0 | 31.6 | 40.1 |
| Transgirls: identity neither | 276 | 13.8 | 38.5 | 46.3 | 29.2 | 34.6 | 41.8 |
| Transgirls: identity varies | 600 | 17.7 | 37.6 | 46.2 | 23.9 | 32.8 | 41.1 |
| Transboys: identity boy | 822 | 9.12 | 56.4 | 72.9 | 46.3 | 52.2 | 63.7 |
| Transboys: identity both | 725 | 10.3 | 63.6 | 78.3 | 52.9 | 56.1 | 66.0 |
| Transboys: identity neither | 1359 | 6.84 | 70.3 | 84.5 | 56.7 | 65.4 | 72.4 |
| Transboys: identity varies | 2401 | 7.41 | 65.4 | 83.5 | 53.6 | 58.6 | 69.0 |
| **P-value^a^** |  | <.0001 | <.0001 | <.0001 | <.0001 | <.0001 | <.0001 |
| ^a^ P-value for heterogeneity between groups. | | | | | | | |

| **Table S2**. Proportions (%) of those being bullied weekly or having poor mental health according to six selected indicators in categories according to school level and gender identity (n= 152 880). | | | | | | | |
| --- | --- | --- | --- | --- | --- | --- | --- |
| **School level and**  **gender identity** | **n** | **Bullied weekly (%)**  **n=152 880** | **Average or bad self-perceived health (%)**  **n=151 732** | **Worries about own mood (%)**  **n=144 659** | **Generalized anxiety**  **(%)**  **n=149 320** | **Depressive symptoms** **(%)**  **n=150 634** | **Social anxiety**  **(%)**  **n=150 881** |
| *8th, and 9th graders from comprehensive school* | | |  |  |  |  |  |
| All | 85927 | 4.97 | 25.4 | 36.1 | 18.9 | 20.9 | 35.0 |
| Cisgirls | 41738 | 4.65 | 31.5 | 50.8 | 28.0 | 28.0 | 46.2 |
| Cisboys | 39906 | 4.47 | 15.3 | 16.0 | 6.32 | 9.70 | 20.1 |
| Transfeminine youth | 831 | 20.5 | 36.7 | 46.3 | 25.3 | 32.1 | 40.4 |
| Transmasculine youth | 3452 | 10.9 | 64.7 | 79.9 | 53.4 | 59.8 | 69.8 |
| P-value^a^ |  | <.0001 | <.0001 | <.0001 | <.0001 | <.0001 | <.0001 |
|  |  |  |  |  |  |  |  |
| *1st and 2nd graders from general upper secondary school* | | | |  |  |  |  |
| All | 46329 | 0.99 | 26.4 | 46.9 | 21.5 | 22.0 | 37.1 |
| Cisgirls | 26423 | 0.90 | 30.4 | 59.8 | 29.3 | 26.8 | 45.0 |
| Cisboys | 18224 | 0.95 | 17.4 | 24.4 | 7.73 | 12.1 | 23.2 |
| Transfeminine youth | 312 | 7.37 | 39.9 | 59.4 | 28.4 | 37.5 | 51.0 |
| Transmasculine youth | 1370 | 1.75 | 65.5 | 86.4 | 52.3 | 58.2 | 66.7 |
| P-value^a^ |  | <.0001 | <.0001 | <.0001 | <.0001 | <.0001 | <.0001 |
|  |  |  |  |  |  |  |  |
| *1st and 2nd graders from* *vocational upper secondary school* | | | |  |  |  |  |
| All | 20624 | 2.37 | 27.9 | 34.4 | 16.6 | 18.2 | 29.6 |
| Cisgirls | 8360 | 2.46 | 38.7 | 55.1 | 28.8 | 27.9 | 42.9 |
| Cisboys | 11605 | 2.11 | 18.3 | 16.7 | 6.02 | 9.25 | 18.2 |
| Transfeminine youth | 174 | 10.3 | 43.9 | 44.5 | 30.2 | 34.3 | 38.7 |
| Transmasculine youth | 485 | 4.12 | 66.0 | 78.5 | 53.9 | 55.7 | 66.3 |
| P-value^a^ |  | 0.0003 | <.0001 | <.0001 | <.0001 | <.0001 | <.0001 |
| ^a^ P-value for heterogeneity between groups. | | | | | | | |

| **Table S3.** Multivariate ^a^ adjusted odds of poor health according to selected indicators between categories of experiences of being bullied in gender identity groups. | | | | | | | | | | | | | | | |
| --- | --- | --- | --- | --- | --- | --- | --- | --- | --- | --- | --- | --- | --- | --- | --- |
|  |  | | | **Cisgender** | | | | |  | **Transgender** | | | | |  |
| **Poor health indicators** | **All**  **n=152880** | |  | **Cisgirls**  **n= 76521** | |  | **Cisboys**  **n= 69735** | |  | **Transfeminine youth**  **n= 1317** | |  | **Transmasculine youth**  **n= 5307** | |  |
|  | **N/n** | **OR (95% CI)** | | **N/n** | **OR (95% CI)** | | **N/n** | **OR (95% CI)** | | **N/n** | **OR (95% CI)** | | **N/n** | **OR (95% CI)** | |
| ***Average or bad self-perceived health*** | |  | |  |  | |  |  | |  |  | |  |  | |
| Experiences of being bullied | 37122/142622 |  | | 23040/72510 |  | | 10402/63965 |  | | 453/1168 |  | | 3227/4979 |  | |
| No (reference) | 27520/117976 | 1 | | 17177/59587 | 1 | | 7936/54202 | 1 | | 280/758 | 1 | | 2127/3429 | 1 | |
| Less frequently | 7508/19995 | 2.02 (1.96-2.09) | | 4704/10720 | 1.93 (1.85-2.02) | | 1889/7870 | 1.96 (1.85-2.08) | | 97/239 | 1.23 (0.91-1.67) | | 818/1166 | 1.47 (1.27-1.71) | |
| At least once a week | 2094/4651 | 2.67 (2.52-2.84) | | 1159/2203 | 2.69 (2.47-2.94) | | 577/1893 | 2.62 (2.36-2.91) | | 76/171 | 1.39 (0.97-1.99) | | 282/384 | 1.73 (1.35-2.20) | |
| P for heterogeneity |  | <.0001 | |  | <.0001 | |  | <.0001 | |  | 0.13 | |  | <.0001 | |
| P for gender identity interaction |  |  | |  |  | |  |  | |  |  | |  | <.0001 | |
|  |  |  | |  |  | |  |  | |  |  | |  |  | |
| ***Worries about own mood*** |  |  | |  |  | |  |  | |  |  | |  |  | |
| Experiences of being bullied | 54213/137030 |  | | 38485/70428 |  | | 11164/60556 |  | | 554/1123 |  | | 4010/4923 |  | |
| No (reference) | 40561/112980 | 1 | | 29385/57705 | 1 | | 8172/51168 | 1 | | 340/728 | 1 | | 2664/3379 | 1 | |
| Less frequently | 10891/19481 | 2.55 (2.47-2.64) | | 7433/10535 | 2.57 (2.45-2.69) | | 2313/7553 | 2.76 (2.60-2.92) | | 121/229 | 1.48 (1.09-2.03) | | 1024/1164 | 2.21 (1.80-2.70) | |
| At least once a week | 2761/4569 | 3.12 (2.93-3.32) | | 1667/2188 | 3.54 (3.19-3.91) | | 679/1835 | 3.62 (3.27-4.02) | | 93/166 | 1.68 (1.16-2.42) | | 322/380 | 1.72 (1.28-2.32) | |
| P for heterogeneity |  | <.0001 | |  | <.0001 | |  | <.0001 | |  | 0.004 | |  | <.0001 | |
| P for gender identity interaction |  |  | |  |  | |  |  | |  |  | |  | <.0001 | |
|  |  |  | |  |  | |  |  | |  |  | |  |  | |
| ***Generalized anxiety*** |  |  | |  |  | |  |  | |  |  | |  |  | |
| Experiences of being bullied | 27437/140488 |  | | 20401/71408 |  | | 4121/63033 |  | | 303/1147 |  | | 2612/4900 |  | |
| No (reference) | 19185/116289 | 1 | | 14684/58703 | 1 | | 2721/53460 | 1 | | 153/748 | 1 | | 1627/3378 | 1 | |
| Less frequently | 6258/19633 | 2.49 (2.40-2.58) | | 4508/10538 | 2.31 (2.21-2.42) | | 960/7717 | 2.92 (2.69-3.17) | | 75/230 | 2.13 (1.51-2.99) | | 715/1148 | 1.83 (1.59-2.11) | |
| At least once a week | 1994/4566 | 4.09 (3.84-4.35) | | 1209/2167 | 3.91 (3.58-4.27) | | 440/1856 | 6.12 (5.43-6.91) | | 75/169 | 3.00 (2.04-4.42) | | 270/374 | 2.86 (2.25-3.63) | |
| P for heterogeneity |  | <.0001 | |  | <.0001 | |  | <.0001 | |  | <.0001 | |  | <.0001 | |
| P for gender identity interaction |  |  | |  |  | |  |  | |  |  | |  | <.0001 | |
|  |  |  | |  |  | |  |  | |  |  | |  |  | |
| ***Depressive symptoms*** | |  | |  |  | |  |  | |  |  | |  |  | |
| Experiences of being bullied | 29666/141715 |  | | 19867/72045 |  | | 6474/63544 |  | | 398/1168 |  | | 2927/4958 |  | |
| No (reference) | 20978/117219 | 1 | | 14281/59223 | 1 | | 4597/53831 | 1 | | 224/758 | 1 | | 1876/3407 | 1 | |
| Less frequently | 6604/19862 | 2.33 (2.25-2.41) | | 4392/10629 | 2.20 (2.11-2.30) | | 1348/7827 | 2.41 (2.25-2.58) | | 90/238 | 1.61 (1.18-2.21) | | 774/1168 | 1.60 (1.38-1.84) | |
| At least once a week | 2084/4634 | 3.74 (3.52-3.98) | | 1194/2193 | 3.69 (3.38-4.03) | | 529/1886 | 4.34 (3.89-4.85) | | 84/172 | 2.30 (1.59-3.32) | | 277/383 | 2.08 (1.64-2.64) | |
| P for heterogeneity |  | <.0001 | |  | <.0001 | |  | <.0001 | |  | <.0001 | |  | <.0001 | |
| P for gender identity interaction |  |  | |  |  | |  |  | |  |  | |  | <.0001 | |
|  |  |  | |  |  | |  |  | |  |  | |  |  | |
| ***Social anxiety*** |  |  | |  |  | |  |  | |  |  | |  |  | |
| Experiences of being bullied | 49784/141942 |  | | 32782/72255 |  | | 13098/63545 |  | | 495/1167 |  | | 3409/4975 |  | |
| No (reference) | 37715/117458 | 1 | | 25316/59407 | 1 | | 9841/53865 | 1 | | 290/761 | 1 | | 2268/3425 | 1 | |
| Less frequently | 9486/19859 | 1.96 (1.90-2.02) | | 6029/10654 | 1.73 (1.66-1.81) | | 2486/7803 | 2.21 (2.10-2.33) | | 112/235 | 1.68 (1.24-2.27) | | 859/1167 | 1.39 (1.19-1.61) | |
| At least once a week | 2583/4625 | 2.68 (2.52-2.85) | | 1437/2194 | 2.49 (2.27-2.73) | | 771/1877 | 3.24 (2.94-3.57) | | 93/171 | 2.18 (1.52-3.12) | | 282/383 | 1.37 (1.08-1.75) | |
| P for heterogeneity |  | <.0001 | |  | <.0001 | |  | <.0001 | |  | <.0001 | |  | <.0001 | |
| P for gender identity interaction |  |  | |  |  | |  |  | |  |  | |  | <.0001 | |
| Abbreviations: CI, confidence interval; n, individuals in the category; N, cases in the category; OR, odds ratio. | | | | | | | | | | | | | | | |
| ^a^ Adjusted for age (continuous), grade, maternal education (low/other), family’s economic situation (poor/other), living situation (placed outside home/other), and origin (foreign/other). | | | | | | | | | | | | | | | |
